# Coupling the Diffusive Transport and Langmuir–Hinshelwood Reaction Kinetics for Kinetic Model Discrimination: A New Insight from an Old Concept

**DOI:** 10.3390/e28050552

**Published:** 2026-05-14

**Authors:** Oleksii Zhokh, Peter Strizhak

**Affiliations:** L.V. Pisarzhevskii Institute of Physical Chemistry of National Academy of Sciences of Ukraine, Nauky Avenue, 31, 03028 Kyiv, Ukraine; pstrizhak@hotmail.com

**Keywords:** effectiveness factor, Langmuir–Hinshelwood kinetics, kinetic model discrimination, diffusion

## Abstract

The overall rate of a heterogeneous catalytic process may be limited by the rate of a chemical transformation on the catalyst surface, reactant transport to either external or internal catalyst surface, or by the interplay between these factors. In this paper, we consider each case concerning the influence of diffusion limitations on the overall process rate. The internal, external, and overall effectiveness factors are obtained for various Langmuir–Hinshelwood kinetic rate equations and catalyst shapes via numerical simulations. It is shown that different kinetic rate equations provide an equally good description of the experimental data obtained under reaction-rate control. In contrast, the internal, external, and overall effectiveness factors may obey dissimilar trends for various kinetic rate equations. The obtained findings are of practical interest since the external and internal diffusion limitations can be achieved by simply changing the feed flow rate in a chemical reactor, catalyst particle size, or temperature increase. Therefore, the presented simulations deliver an easy and comprehensive tool for kinetic model discrimination based on the comparison of the overall effectiveness factors derived in the frame of various rate equations with the experimental one. This result represents a new utilization of an old concept, which is the effectiveness factor, for the selection between the plausible kinetic models.

## 1. Introduction

A heterogeneous catalytic process typically includes bulk reactant transport from the inlet flow to the outer catalyst surface, reactant diffusion from the catalyst boundary to the internal catalyst surface, and adsorption of a reacting species accompanied by its chemical transformation on a catalyst surface. Each of these steps may affect the overall rate of a process. During the design and development of a heterogeneous catalyst and chemical reactor, the elimination of the effect of transport steps on the overall process rate is required to increase the productivity of a process [1]. Nevertheless, many commercially important processes are operated under the conditions of either external or internal diffusion control [2], e.g., ammonia synthesis [3], Fischer–Tropsch process [4], hydrodesulfurization, and hydrodenitrogenation [5]. Therefore, accounting for the effect of the diffusion limitations is an important task for a correct description of the heterogeneous catalytic chemical reactors.

Correct modeling of a chemical reactor also needs the selection of an appropriate kinetic rate equation. The problem is that several rate equations based on different kinetic schemes may simultaneously quantify the intrinsic reaction rate [6]. However, considering various rate equations for the same process and catalyst leads to considerably different results for both technological design [7] and product price [8]. For instance, merely changing the kinetic model may reduce the cost of methanol production from 885 to 801 € per ton [9]. To this end, selection between the rival rate equations is strongly required for optimal process construction and techno-economic assessments.

In the field of heterogeneous catalysis, various strategies to discriminate between the competing kinetic models have been reported. The corresponding tools involve model-based experimental design [10], the analysis of the fitting quality of the experimental data [11], evaluation of methods adopted for parameter estimation [12], and statistical testing of the fitted kinetic parameters [13]. A computational technique for selecting the most relevant kinetic model without human intervention based on the statistical analysis of the process parameters has been introduced [14]. Ab initio calculations of the adsorption energies may successfully be used to select between the plausible Langmuir–Hinshelwood kinetic models [15]. It is possible to reduce the reaction mechanisms and the relevant kinetic models using the kinetic isotope effect [16]. Spectroscopic analysis of the reaction intermediates adsorbed on the catalyst surface also provides evidence of the possible reaction mechanism so that inappropriate kinetic schemes can be excluded from consideration [17]. The influence of the intraparticle diffusion limitations on the process rate may also shed light on the applicability of a particular kinetic model [18].

The effect of the transport limitations in heterogeneous catalysis is usually quantified using the effectiveness factor. The latter represents the ratio between the process rate under the influence of internal and/or external diffusion limitations and the intrinsic reaction rate [19]. Investigations of the effectiveness factor have remained a hot topic for decades. The impact of various factors on the internal effectiveness factor has been explored, e.g., different kinetic rate equations [20], non-isothermal conditions [21], dynamic conditions [22], non-Fickian diffusion [23], external mass transfer resistance [24], and multistep and reversible reactions [25,26]. However, the investigations of the external and overall effectiveness factors, except for the first-order and power-law reaction kinetics [27], are less ubiquitous.

The majority of the approaches currently used for kinetic model discrimination are typically based on the statistical analysis of the fitting and measured kinetic constants. However, the statistical analysis does not reflect the real physics of the process. Therefore, physically based tools should be established for the discrimination purpose. In this paper, we investigate the effectiveness factor of a heterogeneous catalytic process theoretically. Three different scenarios are considered concerning intraparticle diffusion limitations, external mass transfer limitations, and the complex interplay between external and internal diffusion limitations. In each scenario, the effectiveness factor typically follows different trends depending on the expression of the Langmuir–Hinshelwood kinetic rate equation. Keeping in mind that various rate equations sometimes provide almost identical descriptions of the reaction rate, the presented simulations may be used for the experimental data analysis to select between the rival kinetic models.

## 2. Materials and Methods

### 2.1. Preliminaries

**Figure 1:**
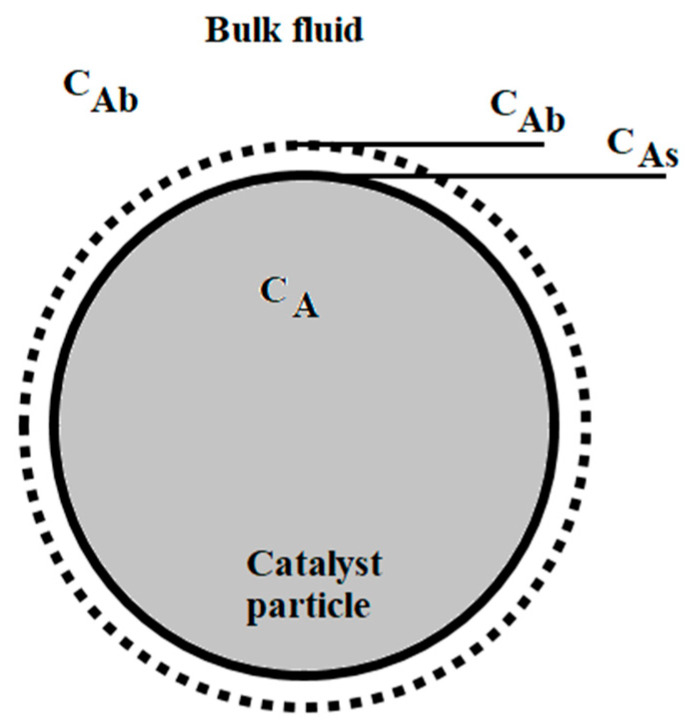
A sketch of a catalyst particle.

Herein, we provide some introductory textbook information regarding the effect of diffusion limitations on the rate of a heterogeneous catalytic process. Under diffusion limitations, three different concentrations may be involved in heterogeneously catalyzed chemical conversion. Consider *C_A_* being the reactant concentration in the catalyst particle, m^3^/m^3^, *C_Ab_* being the reactant concentration in the bulk fluid, m^3^/m^3^, and *C_As_* being the reactant concentration at the outer catalyst surface, m^3^/m^3^. A sketch of the porous solid catalyst particle with the corresponding concentrations is shown in Figure 1. The following scenarios may arise: (i) the process rate is unaffected by reactant diffusion and equals the intrinsic reaction rate, i.e., *C_A_* = *C_As_* = *C_Ab_*, (ii) the process rate is limited by reactant diffusion inside the catalyst particle, i.e., *C_A_* < *C_As_* = *C_Ab_*, (iii) the process rate is limited by reactant diffusion to the outer surface of a catalyst particle, i.e., *C_A_* = *C_As_* < *C_Ab_*, and (iv) the process rate is limited by both reactant diffusion inside the catalyst particle and to the outer particle’s surface, i.e., *C_A_* < *C_As_* < *C_Ab_*. Each scenario is theoretically addressed in full detail in Section 2.2, Section 2.3, Section 2.4 and Section 2.5.

### 2.2. Intrinsic Reaction Rate

Consider an irreversible chemical reaction 2*A* → *B* + *E*, where *A* is the reactant and *B* and *E* are the reaction products. The reaction is heterogeneously catalyzed by a porous solid. The catalyst particles may be of slab, spherical, or cylindrical geometry. The reaction mechanism obeys the Langmuir–Hinshelwood kinetics. The reaction rate is unaffected by the mass transfer limitations. Contemplate the following general rate equation describing the consumption of reactant *A* derived under Langmuir–Hinshelwood formalism.

(1) $$-r\left(C_{A}\right)=k\cdot \frac{{\left(K\cdot C_{A}\right)}^{m}}{{\left(1+K\cdot C_{A}\right)}^{n}},$$

where −*r* is reaction rate, m^3^/(m^3^ × s), *K* is the adsorption equilibrium constant, dimensionless, *k* is reaction rate constant, m^3^/(m^3^ × s), and *m* and *n* are some constants, the values of which depend on the kinetic model.

In Equation (1), the terms concerning the reaction products are neglected for the sake of computational simplicity. This equation with various *m* and *n* is often utilized for a description of the reaction rates of many processes, e.g., conversion of different alcohols [28,29,30]. As it is mentioned above, *C_As_* and *C_Ab_* can be used in Equation (1) instead of *C_A_*. Under the influence of the mass transfer limitations, Equation (1) will also work. Consider the case of the presence of both external and internal mass transfer limitations. In this case, *r*(*C_A_*) ≠ *r*(*C_As_*) ≠ *r*(*C_Ab_*) and *C_A_* ≠ *C_As_* ≠ *C_Ab_*. In the experiment, only *C_Ab_* can be measured experimentally. Therefore, *r*(*C_A_*) and *r*(*C_As_*) should be evaluated in terms of *C_Ab_*. This requires the diffusion modeling of the process kinetics. The corresponding modeling is typically treated as the solution of the steady-state reaction–diffusion problem in the form of the effectiveness factor.

### 2.3. Internal Effectiveness

Provided the reaction rate is higher than the diffusion rate in the small catalyst pores, the reactant concentration inside the catalyst particle is lower than the reactant concentration at the outer surface of the catalyst particle. The effect of the intraparticle diffusion limitations is quantified by the internal effectiveness factor. By definition, the effectiveness factor reflects the ratio between the actual rate of a heterogeneous catalytic process (i.e., under diffusion limitations) and the process rate in the absence of the diffusion resistance (i.e., intrinsic reaction rate). For the isothermal steady-state conditions, the reaction–diffusion process is given by the following problem.

(2) $$D_{e}\cdot \left(\frac{d^{2}C_{A}}{dx^{2}}+\frac{\delta }{x}\cdot \frac{dC_{A}}{dx}\right)=-r\left(C_{A}\right),\;x\in \left[0,L\right]$$

(3) $$C_{A}=C_{As},\;x=L$$

(4) $$\frac{dC_{A}}{dx}=0,\;x=0$$

where *D_e_* is the effective diffusion coefficient, m^2^/s, *x* is the space coordinate, m, *δ* is the shape factor, dimensionless (*δ* = 0, 1, and 2 for planar, cylindrical, and spherical catalyst shapes, respectively), and *L* is the half-size of a catalyst particle, m.

To solve Equations (2)–(4) with respect to the internal effectiveness factor, the dimensionless variables are introduced so that *u* = *C_A_*/*C_As_*, *s* = *x*/*L*, *σ* = *K* × *C_As_*, and the Thiele modulus *φ* is defined by

(5) $$\varphi =\sqrt{\frac{K\cdot k\cdot L^{2}}{D_{e}}\cdot \frac{1}{{\left(1+\sigma \right)}^{2}}}.$$

The dimensionless function *r*(*u*) for each rate equation is collected in Table 1. Thereafter, Equations (2)–(4) are rearranged into a dimensionless form.

(6) $$\frac{d^{2}u}{ds^{2}}+\frac{\delta }{s}\cdot \frac{du}{ds}=\varphi^{2}\cdot r\left(u\right)$$

(7) u=1, s=1

(8) $$\frac{du}{ds}=0,\;s=0$$

The internal effectiveness factor *η_int_* is defined by

(9) $$\eta_{int}=\frac{1+\delta }{\varphi^{2}}\cdot \frac{du}{ds},\;s=1.$$

The solution to Equation (9) is obtained numerically using the ‘NDSolve’ operator available within the Wolfram Mathematica 13.1 package. The numeric solutions are derived as the function *η_int_*(*C_As_*), i.e., provide the dependence of the internal effectiveness factor on the reactant concentration at the outer catalyst surface *C_As_*. The latter concentration is used since the reactant concentration inside the catalyst particle *C_A_* is neither initially known nor can easily be measured experimentally. These numeric results are utilized during further considerations concerning the overall effectiveness.

For the sake of modeling, a generic effective diffusion coefficient is utilized. The effective diffusion coefficient may be considered in the frame of molecular Fickian diffusion, Knudsen diffusion, or the interplay between Knudsen and bulk diffusion, i.e., under the Bosanquet relation. Knudsen and bulk diffusion reflect the self-diffusion coefficient. For multi-component mixtures, Fick’s and Maxwell–Stefan diffusion models may be used [31].

### 2.4. External Effectiveness

If the rate-limiting step of a heterogeneous catalytic process is the diffusion from the fluid phase to the outer surface of a catalyst, the process rate equals the reactant flux to the outer catalyst surface [19].

(10) $$k_{f}\cdot a_{m}\cdot \left(C_{Ab}-C_{As}\right)=-r\left(C_{As}\right)=\eta_{ext}\cdot \left(-r\left(C_{Ab}\right)\right),$$

where *k_f_* is the mass transfer coefficient, m/s, *a_m_* is the effective interfacial surface area, m^2^/m^3^, and *η_ext_* is the external effectiveness factor, dimensionless.

The external effectiveness factor can be obtained from Equation (10) in two ways. The first approach implies the elimination of *C_As_*, which is neither initially known nor can be easily measured experimentally. The elimination procedure is a usual technique, e.g., for first-order reactions [27]. The second approach is based on the introduction of the dimensionless variables and solving the resulting algebraic equation. This technique is often used for Langmuir–Hinshelwood or power-law kinetics [32,33] because the analytic elimination for these types of reaction kinetics may be too complicated. Consider the dimensionless variables *U* = *C_As_*/*C_Ab_*, *β* = (*K* × *C_Ab_*)^−1^, *γ* = *k_f_* × *a_m_* × (*k* × *K*)^−1^, and the second Damköhler number *Da_II_* expressed as:

(11) $$Da_{II}=\frac{k}{\frac{k_{f}\cdot a_{m}}{K}+k_{f}\cdot a_{m}\cdot C_{Ab}}$$

Using the dimensionless variables, the *k_f_* × *a_m_* × (*C_Ab_* − *C_As_*) = −*r*(*C_As_*) part of Equation (10) for various *m* and *n* is rewritten as follows:

(12) $$\frac{\left(1-\gamma \cdot Da_{II}\right)\cdot \left(1-U\right)}{Da_{II}\cdot \beta^{n-m}}=\frac{U^{m}}{{\left(\beta +U\right)}^{n}}$$

In this paper, we utilize the elimination procedure to determine the dependence of the external effectiveness factor on the reactant concentration in the bulk phase. Regarding the dimensionless variables, Equation (11) will be used to analyze the dependence of the external efficiency on the second Damköhler number. The latter analysis is a typical strategy in chemical engineering.

The reactant concentration in the bulk fluid phase *C_Ab_* is known from the experiment. Therefore, the elimination of *C_As_* yields the dependence of the external effectiveness factor on *C_Ab_*. For instance, the corresponding elimination may be achieved by solving analytically the equality *k_f_* × *a_m_* × (*C_Ab_* − *C_As_*) = −*r*(*C_As_*) for *C_As_*. The obtained expressions for *C_As_* in terms of *C_Ab_*, mass transfer coefficient, and kinetic constants are substituted in the equality −*r*(*C_As_*) = *η_ext_* × (−*r*(*C_Ab_*)) so that *η_ext_*(*C_Ab_*) is obtained. A similar elimination procedure for *C_Ab_* may also be applied to derive *η_ext_*(*C_As_*). However, the latter has little practical significance due to the impossibility of *C_As_* experimental measurement. The elimination procedure was completed using the ‘Solve’ operator in the Wolfram Mathematica 13.1 package. The closed-form expressions for *η_ext_*(*C_Ab_*) and *η_ext_*(*Da_II_*) are not presented here due to their analytic complexity.

### 2.5. Overall Effectiveness

In the presence of external and internal diffusion limitations, the overall process rate follows the relation [34]

(13) $$k_{f}\cdot a_{m}\cdot \left(C_{Ab}-C_{As}\right)=\eta_{int}\cdot \left(-r\left(C_{As}\right)\right)=\eta_{O}\cdot \left(-r\left(C_{Ab}\right)\right),$$

where *η_O_* is the overall effectiveness factor, dimensionless.

Under Equation (13), the explicit elimination procedure is possible only for *C_As,_* provided the analytic expression for *η_int_* is available. However, *η_O_*(*C_Ab_*) can be obtained from the numerical simulations. With the knowledge about the kinetic constants and mass transfer parameters, *C_Ab_* may be evaluated from the relation *k_f_* × *a_m_* × (*C_Ab_* − *C_As_*) = *η_int_* × (−*r*(*C_As_*)) for exact values of *C_As_*, i.e., solving the backward problem. Knowing the value of *C_Ab_* for each *C_As_*, the overall effectiveness is computed via utilization of Equation (10). The corresponding procedure is performed by applying the ‘NSolve’ operator ready for use in the Wolfram Mathematica 13.1 package.

Substituting Equations (5) and (9), which are the definitions of Thiele modulus and internal effectiveness factor, respectively, in Equations (13), and doing some mathematics, one obtains

(14) $$\frac{Bi_{m}\cdot a_{m}\cdot K\cdot k\cdot L\cdot \left(C_{Ab}-C_{As}\right)}{\left(1+\delta \right)\cdot {\left(1+K\cdot C_{As}\right)}^{2}}=\frac{du}{ds}{|}_{s=1}\cdot \left(-r\left(C_{As}\right)\right)=\eta_{O}\cdot \left(-r\left(C_{Ab}\right)\right),$$

where *Bi_m_* is the mass transfer Biot number (also called modified Sherwood number). It is given by [35]

(15) $$Bi_{m}=\frac{k_{f}\cdot L}{D_{e}}.$$

Equation (14) represents the relationship between the mass transfer Biot number and the overall effectiveness factor. Therefore, it is convenient to scrutinize the dependence of the external efficiency on the mass transfer Biot number.

### 2.6. Experimental

To demonstrate the behavior of the effectiveness factor for various kinetic equations, we adopt the exemplary experimental data concerning the methanol dehydration to dimethyl ether over a commercial γ-alumina catalyst under reaction-rate control. The rate of the corresponding process under reaction-rate control may be described by Equation (1) with various *m* and *n*. The relevant kinetic data were taken from the literature [36]. The experimental data are represented as the rate of the methanol consumption in moles per unit catalyst mass per unit time versus methanol mole fraction in the reaction mixture. The experimental data set was obtained under the following conditions. The reaction temperature was kept at 433 K. The catalyst particle size was less than 0.5 mm. The WHSV ranged between 2.4 and 42.9 h^−1^ × (g/g). The kinetic experiment was conducted in a fixed-bed flow reactor. The kinetic constants in Equation (1) with various *m* and *n* were derived from the experimental data fitting. The fitting was performed using the Levenberg–Marquardt algorithm. The objective function aimed to minimize the sum of the squares of residuals. This method is a built-in tool of the ‘NonLinearModelFit’ operator available within the Wolfram Mathematica 13.1 package. In brief, the least square method, also called chi-square minimization, utilizes the following objective function χ^2^:

(16) $$\chi^{2}\left(\theta \right)=\sum_{i=1}^{j}\frac{{\left(y_{i}-f\left(l_{i},\theta \right)\right)}^{2}}{\omega_{i}^{2}},$$

Herein, *y_i_* are the experimental data, *f*(*l_i_*, *θ*) is the model, *l_i_* is the row vector for the *i*-th measurement, and *ω_i_* is the uncertainty. The parameters *θ* that yield the lowest *χ*^2^(*θ*) are treated as the best-fit parameters.

To convert the dimension of the rate constant from mol/(s × g) units into m^3^/(m^3^ × s) units, the fitted rate constants were corrected by the factor of 853 × 1000 × 22.4 × 10^−3^, where 853 is the particle density of the γ-alumina catalyst in kg/m^3^ units, 22.4 × 10^−3^ is the molar volume in m^3^/mol units, and 1000 is the coefficient to rescale g into kg. For the evaluation of the effectiveness factor, the following values of the mass transfer parameters are used: *D_e_* = 1 × 10^−9^ m^2^/s, *k_f_* = 0.05 m/s, *a_m_* = 1 m^2^/m^3^, and *L* = 5 × 10^−3^ m. These values may not directly reflect the real situation that occurs in an industrial chemical reactor for γ-alumina catalyst. However, they allow us to demonstrate the influence of various parameters on the effectiveness factor.

## 3. Results and Discussion

### 3.1. Reaction Kinetics

**Figure 2:**
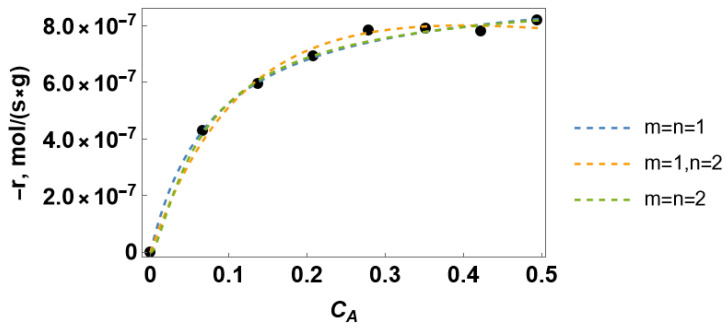
Experimental rate of alcohol conversion (points) fitted by Equation (1) (dashed lines).

The exemplary rate of the alcohol conversion under reaction-rate control fitted by Equation (1) is shown in Figure 2. The reaction rate obeys the classical Langmuir–Hinshelwood trend. The correspondence between the experimental data and the rate equation is equally good for various *m* and *n*. The kinetic constants estimated from the fitting procedure are collected in Table 2. The obtained results demonstrate that an exact kinetic equation cannot be selected as the most relevant one based on the experimental data fitting. A discrimination tool is required to choose the best-fit kinetic model.

### 3.2. Process Efficiency Simulations

#### 3.2.1. Internal Effectiveness

**Figure 3:**
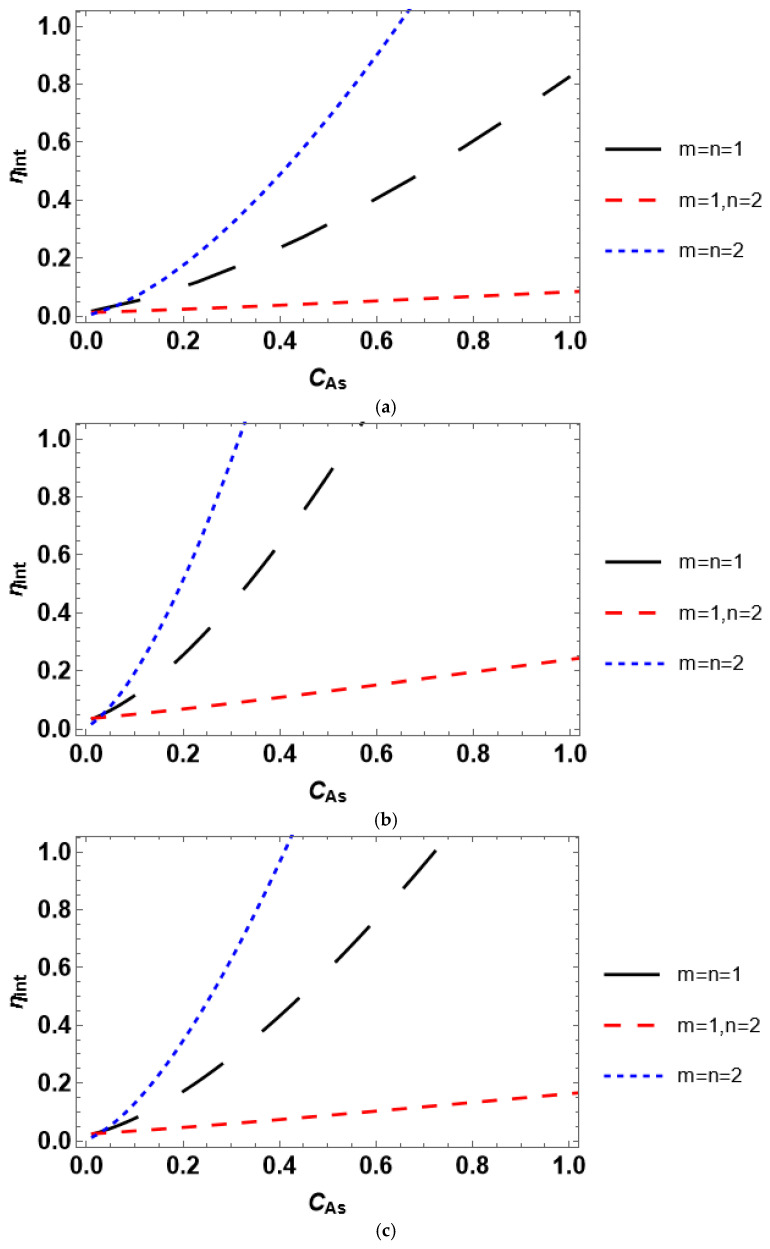
Influence of the reactant concentration on the internal effectiveness factor for a catalyst with (**a**) planar, (**b**) spherical, and (**c**) cylindrical shape.

Herein, we perform the analysis of the impact of the reactant concentration on the internal effectiveness factor, as well as analyze the interconnection between the internal efficiency and the Thiele modulus. Figure 3 presents the concentration dependence of the internal effectiveness factor for various catalyst shapes. The internal effectiveness factor increases with the growth of reactant concentration; however, it experiences significantly different trends concerning the form of the Langmuir–Hinshelwood kinetic rate equation. This situation holds regardless of the considered geometry of a catalyst particle. The internal effectiveness reaches higher values at lower *C_As_* in the following order: sphere > cylinder > slab.

**Figure 4:**
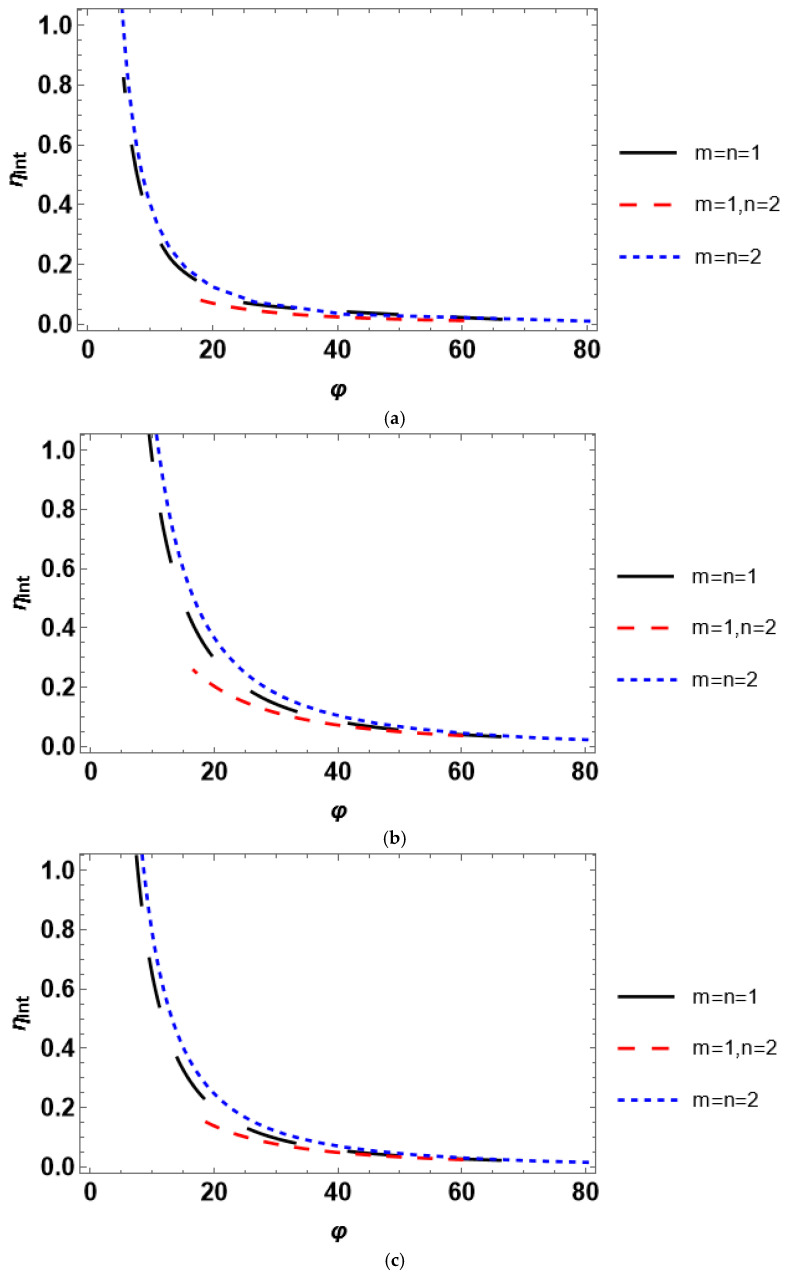
Influence of the Thiele modulus on the internal effectiveness factor for a catalyst with (**a**) planar, (**b**) spherical, and (**c**) cylindrical shape.

The Thiele modulus quantifies the relationship between the reaction and diffusion rates. If *φ* > 1, the reaction rate prevails over the diffusion rate, and vice versa for *φ* < 1. Therefore, intraparticle diffusion affects the process rate provided *φ* > 1. The higher the Thiele modulus, the stronger the intraparticle diffusion limitations. The modeled relationship between the internal effectiveness and Thiele modulus is depicted in Figure 4. The effectiveness factor exhibits a convenient trend implying diminution (or stronger intraparticle diffusion limitations) with an increase in the Thiele modulus for all catalyst geometries. The change in the Thiele modulus is achieved by varying the reactant concentration. The latter corresponds to the mole fraction of a reactant, i.e., it ranges between 0 and 1. To obtain the plots in Figure 4, the values of the concentration between 0.1 and 1.0 with a step size of 0.1 were employed in the numerical solution. The variation in *φ* may also be reached using any other parameter, e.g., catalyst particle size, reaction rate constant, effective diffusivity, and equilibrium constant. In practice, for a certain heterogeneous catalytic system, the catalyst particle size and reactant concentration are the only parameters that can be easily changed during the experiment. It should also be emphasized that varying the Thiele modulus in a different way than changing the reactant concentration does not affect the trends obtained in Figure 4.

Both scientific papers and textbooks typically consider only the effect of the Thiele modulus on the internal effectiveness factor. This is reasonable for establishing the bounds of the reaction-rate control and intraparticle diffusion-controlled regimes of a heterogeneous catalytic process. However, this is almost useless for the discrimination of a kinetic model of the process. According to the data in Figure 4, *η_int_*(*φ*) is quite similar for different kinetic rate equations. In contrast, this is not the case for *η_int_*(*C_As_*), which follow notably dissimilar trends under various kinetic rate equations (Figure 3). Therefore, considering the concentration dependence of the internal effectiveness factor delivers a new and comprehensive tool for selecting between the rival kinetic models.

#### 3.2.2. External Effectiveness

**Figure 5:**
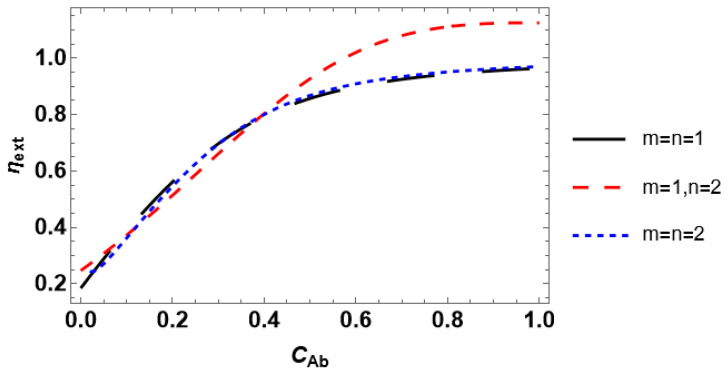
Influence of the reactant concentration on the external effectiveness factor.

The effect of the reactant concentration in the bulk phase on the external effectiveness factor is demonstrated in Figure 5. The external effectiveness factor is almost identical for the kinetic rate equations with *m* = *n*. For all kinetic rate equations, the effectiveness factors nearly coincide in the *C_Ab_* range between 0.02 and 0.45. An increase in bulk reactant concentration governs the growth of the effectiveness factor until it reaches unity (*m* = *n*) or overreaches unity up to approximately 1.15 (*m* =1, *n* = 2). It should be stressed that the external efficiency may be either lower or higher than unity depending on the reaction order [19]. Particularly, *η_ext_* is always greater than unity if the reaction order is negative. Therefore, the attained result concerning *η_ext_* > 1 for *m* =1, *n* = 2 (Figure 5) may be verified by calculating the reaction order *N*. The latter can be calculated from the relation [37]

(17) $$N=C_{A}\cdot \frac{d\ln r\left(C_{A}\right)}{dC_{A}}.$$

**Figure 6:**
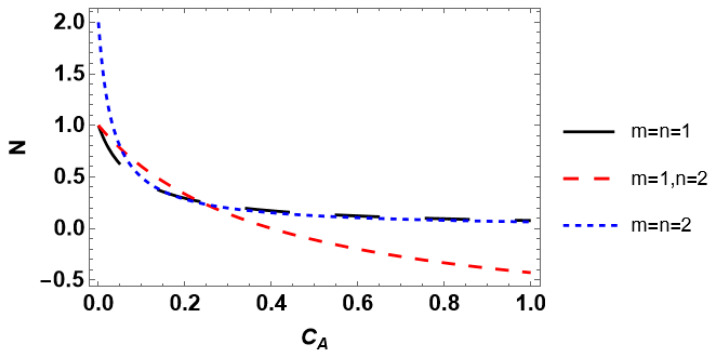
Influence of the reactant concentration on the reaction order.

The plots of the reaction order (Figure 6) obtained under Equation (17) corroborate the observed trends for the effectiveness factor. For *m* = *n*, the reaction order is positive. As a consequence, the external effectiveness factor is lower than unity. In contrast, the reaction order under *m* = 1, *n* = 2 becomes negative at *C_A_* ≈ 0.40, and the external effectiveness is greater than 1 at *C_Ab_* ≈ 0.55. The difference between *C_A_* and *C_Ab_* demonstrates how much the external diffusion limits the reaction rate, i.e., *r*(*C_A_* ≈ 0.40) = *r*(*C_Ab_* ≈ 0.55).

**Figure 7:**
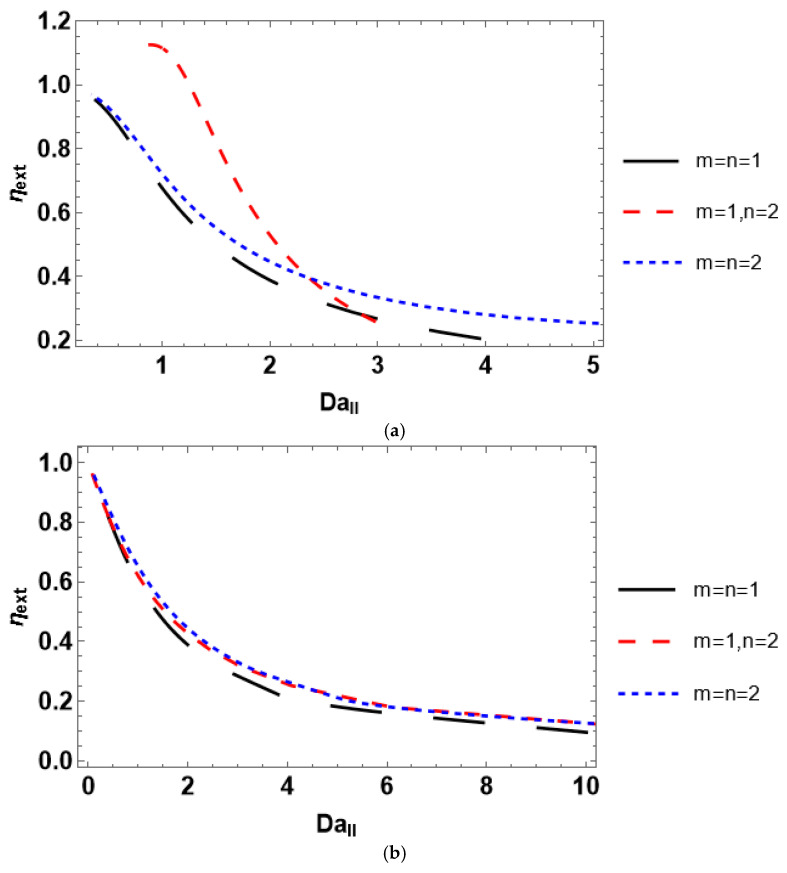
Influence of the second Damköhler number on the external effectiveness factor obtained by varying (**a**) reactant concentration and (**b**) mass transfer coefficient.

The second Damköhler number illustrates the ratio between the chemical reaction rate and interphase mass transfer rate. The observed process rate is defined by the interphase transport and chemical reaction rate for *Da_II_* > 1 and *Da_II_* < 1, respectively [38]. In the experiment, the second Damköhler number may be varied by changing either the bulk reactant concentration or the mass transfer coefficient. The effect of the second Damköhler number on the external effectiveness factor is investigated by plotting the corresponding profiles (Figure 7). The external effectiveness exhibits a decrease with an increase in the Damköhler number, as it should. It is interesting to note that varying the reactant concentration yields considerably different trends of *η_ext_*(*Da_II_*) for various *m* and *n*. In contrast, plotting *η_ext_* against *Da_II_* using fixed *C_Ab_* (*C_Ab_* = 0.1) and miscellaneous values of the mass transfer coefficient gives very similar curves for various *m* and *n*.

The obtained findings indicate that investigating the dependence of the external effectiveness factor on the reactant concentration is required if one aims to perform discrimination between the plausible kinetic rate equations. Considering *η_ext_*(*Da_II_*) for the discrimination purpose, one also needs to alter the reactant concentration since fixing the concentration at a given value may lead to misleading results. However, the discrimination capability in the case of external effectiveness is rather limited compared to internal effectiveness. For some combinations of *m* and *n*, the dependencies of *η_ext_* on *C_Ab_* and *Da_II_* follow almost identical trends. Moreover, a conclusion on the possibility of the kinetic model discrimination based on the external effectiveness factor may be drawn from the preliminary study of the concentration dependence of the reaction order. This can be carried out theoretically using Equation (14) and the corresponding kinetic rate equation, even without experimental measurements. If some kinetic rate equations admit positive reaction order, whereas the other rate equations imply negative reaction order, discrimination based on external effectiveness is principally possible. Therefore, it is necessary to measure the experimental effectiveness. Provided all rate equations are of the same reaction order, scarcely any utilization of the external effectiveness allows the discrimination between the competing rate equations.

#### 3.2.3. Overall Effectiveness

**Figure 8:**
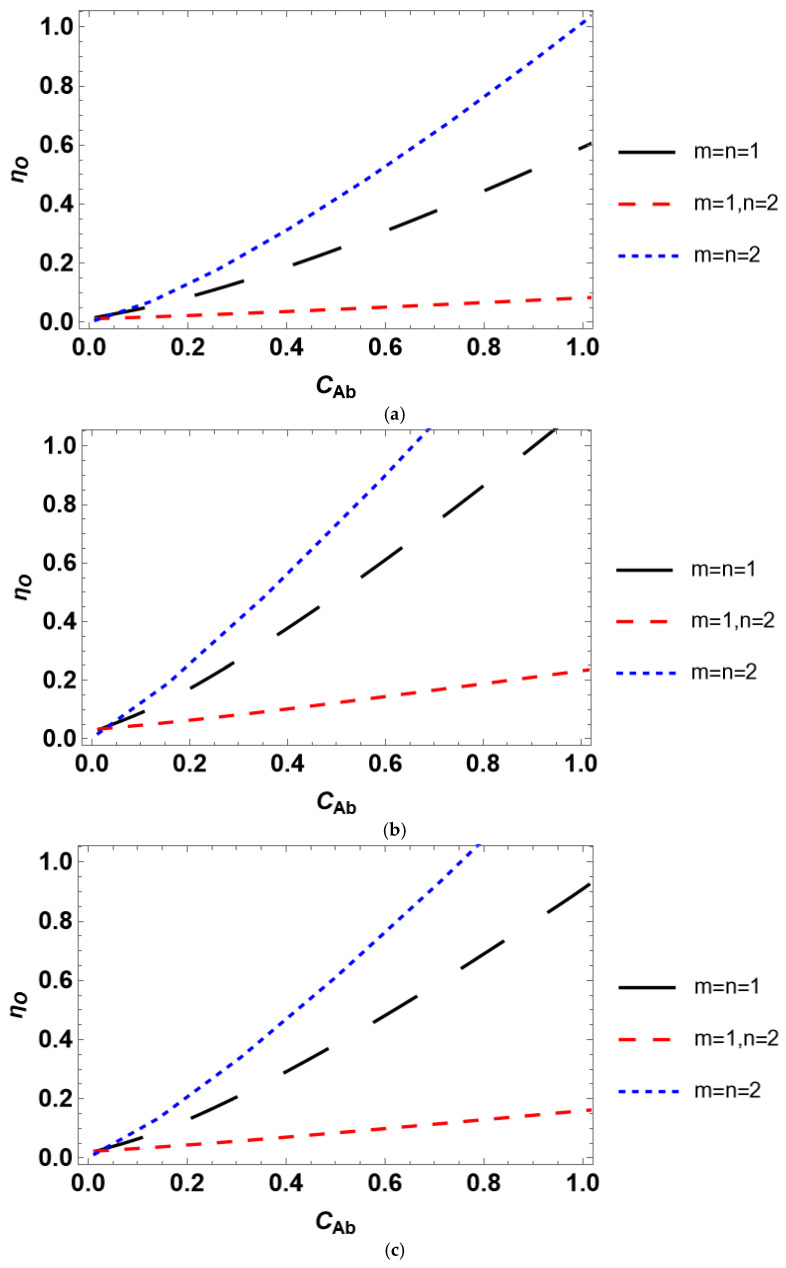
Influence of the reactant concentration on the overall effectiveness factor for a catalyst with (**a**) planar, (**b**) spherical, and (**c**) cylindrical shape.

Accounting for the effect of both internal and external diffusion limitations yields the overall effectiveness factor. The overall efficiency plotted against bulk reactant concentration is presented in Figure 8. *η_O_* increases with the growth in the reactant concentration for all catalyst shapes and kinetic rate equations similar to *η_ext_*. *η_O_*(*C_Ab_*) trends are also significantly different for various rate equations. This may be expected since the overall effectiveness factor includes both internal and external efficiencies. The internal effectiveness significantly deviates for different kinetic rate equations compared to the external effectiveness. The overall effectiveness factor demonstrates a slower growth with an increase in the bulk reactant concentration than the growth of the internal effectiveness factor with an increase in the surface concentration, as follows from the comparison of the data in Figure 3 and Figure 8. This is governed by the effect of external diffusion limitations.

**Figure 9:**
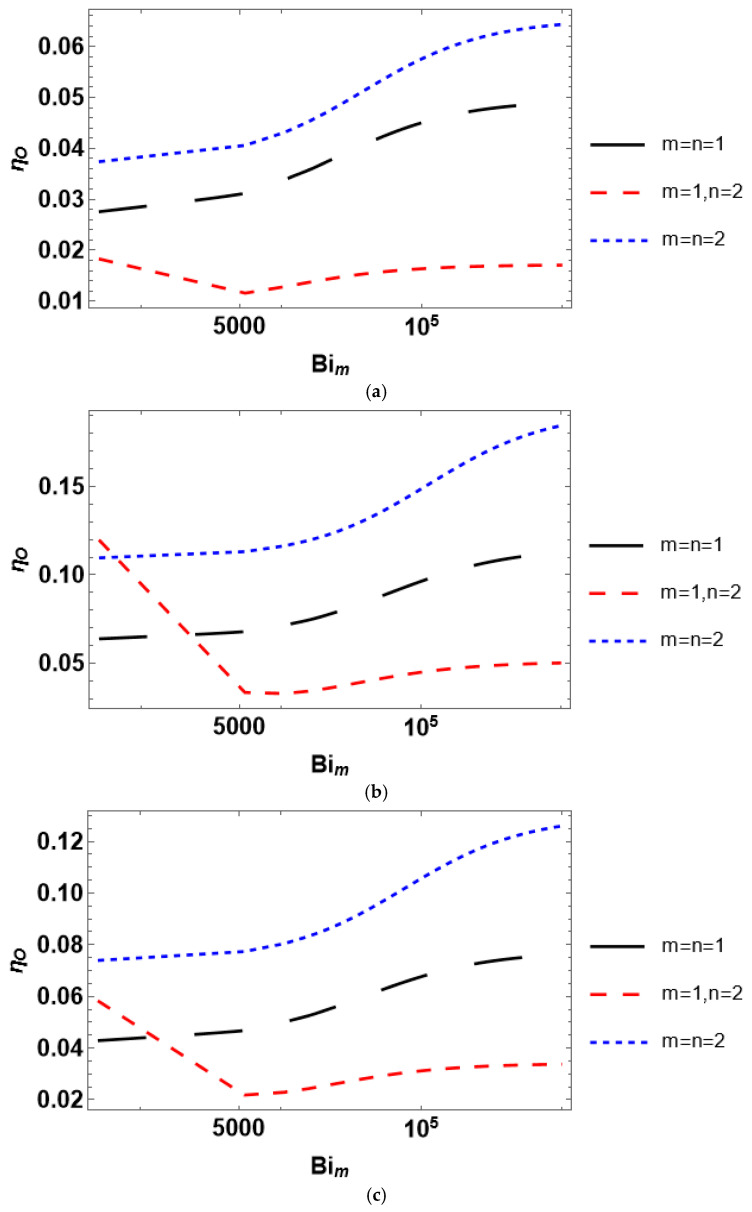
Influence of the mass transfer Biot number on the overall effectiveness factor for a catalyst with (**a**) planar, (**b**) spherical, and (**c**) cylindrical shape.

The overall effectiveness is an interplay between the external and internal diffusion limitations. The relationship between external and internal diffusion rates is quantified using the mass transfer Biot number. By definition, if *Bi_m_* → ∞, the external mass transfer limitations can be neglected. To this end, the overall efficiency is analyzed with respect to the mass transfer Biot number (Figure 9). Since *Bi_m_* is independent of the reactant concentration, the plots in Figure 9 are obtained by varying the mass transfer coefficient and fixing the reactant concentration at *C_As_* = 0.1. The overall effectiveness reaches a plateau as *Bi_m_* approaches infinity, i.e., *η_O_* becomes insensitive to *Bi_m_*. In contrast, at finite values of the Biot number, the overall effectiveness factor indicates strong dependence on *Bi_m,_* implying either rapid growth (for *m* = *n*) or having a local minimum (for *m* ≠ *n*). An interesting observation may be made by comparing the data presented in Figure 3 and Figure 9. The overall effectiveness factor at *Bi_m_* → ∞ (Figure 9) reaches approximately the same values as the internal effectiveness factor at *C_As_* = 0.1 (Figure 3), i.e., *η_O_*(*C_As_* = 0.1, *Bi_m_* → ∞) ≈ *η_int_*(*C_As_* = 0.1). Thus, only internal diffusion limitations are considerable at infinitely large *Bi_m_*.

The overall effectiveness factor behaves conveniently with the mass transfer Biot number. For the kinetic model discrimination, the analysis based on either reactant concentration or *Bi_m_* may be suitable because both *η_O_*(*Bi_m_*) and *η_O_*(*C_Ab_*) differ significantly under various *m* and *n*. It should be emphasized that plotting *η_O_* against *Bi_m_* at different *C_As_* yields qualitatively the same trends as those presented in Figure 9.

### 3.3. Verification of the Numeric Results

**Figure 10:**
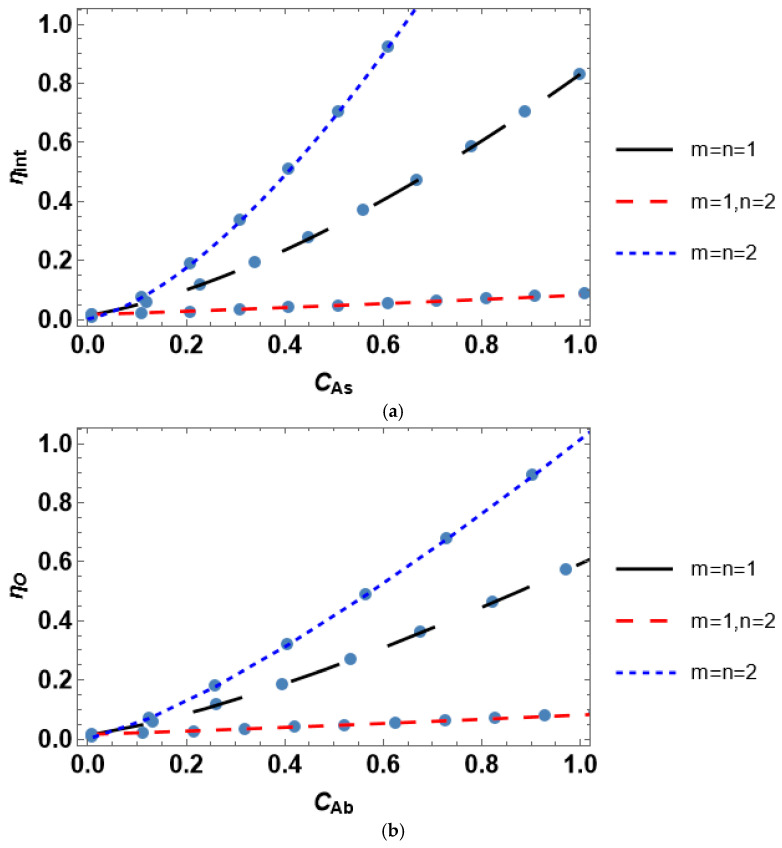
Comparison of the (**a**) internal and (**b**) overall effectiveness factors obtained using the numerical (points) and analytical (dashed lines) solutions.

The internal effectiveness factor is calculated using the numerical solution of the corresponding steady-state reaction–diffusion problem. The overall effectiveness factor is also based on the numeric results concerning the internal effectiveness factor. To verify the numerical computations, we compare the obtained numerical solutions with the analytic solutions presented in the literature. Particularly, the closed-form analytic expressions for the internal effectiveness factor for a catalyst particle of slab geometry are collected in Table 3. The numerical solutions of the internal effectiveness factor and the equations from Table 3 in pictorial form are shown in Figure 10. An essential correspondence between the analytic and numerical solutions is observed. The analytic solutions of the reaction–diffusion problem may also be used instead of the numerical techniques. However, the derivation of the analytic representations of the effectiveness factors in spherical or cylindrical coordinates may be too complicated.

The analytical equations for the internal effectiveness factor, presented in Table 3, are valid under the condition of internal diffusion limitations. The presence of the external mass transfer resistance, i.e., the condition of simultaneous external and internal diffusion limitations, does not affect the corresponding equations. It should also be emphasized that the equations in Table 3 do not reduce to first- or second-order solutions if *K* ≈ 0. The substitution of *K* = 0 in these equations yields 0, since the intrinsic rate −*r*(*C_As_*) = 0 for *K* = 0, according to Equation (1). Morbidelli and Varma [40] demonstrated that the corresponding reduction can be achieved via the substitution of the dimensionless parameter *σ* = 0 in the expression for *r*(*u*).

A situation analogous to that for the internal effectiveness factor is observed if the overall efficiency is considered. The overall effectiveness factors obtained via the substitution of the expressions from Table 3 in Equation (13) exhibit virtually the same dependence on *C_Ab_* as *η_O_* derived using the numerical simulations of the internal effectiveness factor (Figure 10b). Therefore, using the numerical solutions to establish the internal and overall effectiveness factors is an acceptable scenario that provides correct results.

### 3.4. Discrimination Approach

To determine which phenomenon, e.g., reaction, intraparticle diffusion, or external diffusion, is dominating and to predict the behavior of the effectiveness factor, the dimensionless parameters are typically utilized. The dimensionless parameters may be the Thiele modulus, mass transfer Biot number, and second Damköhler number. In this aspect, the results of the simulations obtained in this paper for the dependence of the effectiveness factor on the corresponding dimensionless parameters are generally known. Herein, we highlight a new insight into the role of diffusion limitations in heterogeneous catalysis by exploring the potential branches for discrimination between the rival kinetic models based on the application of the diffusion limitations. To this end, the obtained results are discussed from the point of view that tends to support this fairly new discrimination approach.

Considering dimensionless parameters is reasonable for industrial-scale reactor modeling using certain process conditions and a catalyst with prescribed properties. A particular isothermal reactant–catalyst system is characterized by fixed values of the effective diffusivity and reaction rate constant. The dimensionless parameters are usually varied by changing catalyst particle size (Thiele modulus) or mass transfer coefficient (*Bi_m_* and *Da_II_*). The presented analysis of the internal effectiveness factor as a function of Thiele modulus (Figure 4) and the external effectiveness factor as a function of the second Damköhler number (Figure 7) reveals that the kinetic models can scarcely be discriminated against because *η_int_*(*φ*) and *η_ext_*(*Da_II_*) experience nearly equal trends for different kinetic rate equations. However, the situation is different for the dependence of the overall effectiveness factor on the mass transfer Biot number. *η_O_*(*Bi_m_*) plots are markedly unequal for diverse rate equations even at identical reactant concentrations (Figure 9). In contrast, accounting for the dependence of the effectiveness factor on the reactant concentration gives disparate tendencies. Therefore, a simple lab-scale approach is to analyze the process efficiency by varying the reactant concentration, which is easy to achieve experimentally.

A conclusion on the applicability of a particular kinetic model for a description of the rate of a heterogeneous catalytic process may be drawn following a simple and heuristic procedure. The first step involves an investigation of the influence of reactant concentration on the intrinsic reaction rate and its fitting by the corresponding kinetic rate equation to estimate the kinetic constants. The second step implies investigation of the concentration dependence of the process rate under the influence of internal, external, or both internal and external diffusion limitations. The impact of the internal diffusion limitations on the process rate may be achieved by increasing the catalyst particle size or the reaction temperature. Increasing the reaction temperature requires recalculation of the kinetic constants using activation energy and adsorption heat to evaluate the actual intrinsic reaction rate at the elevated temperature. The effect of external diffusion limitations on the process rate becomes significant with a decrease in the total feed flow rate or catalyst particle size. Either the absence or presence of the diffusion limitations might be controlled via evaluation of the mass transfer criteria, which may be the Thiele modulus, Weisz–Prater and Mears criteria for internal diffusion limitations, and Carberry and Damköhler numbers for external diffusion limitations. The presence of both internal and external diffusion limitations may be verified from the mass transfer Biot number. In the third step, the process rate observed in the presence of diffusion limitations is compared to the intrinsic reaction rate to evaluate the experimental effectiveness factor as a function of reactant concentration. Finally, the experimental effectiveness factor is compared to the theoretical ones obtained from either numerical simulations or analytic solutions for various kinetic rate equations. A good correspondence between the experimental and theoretical effectiveness factor for a certain kinetic model indicates that such a model is acceptable in the scenario under consideration.

The use of the discrimination approach may imply some limitations. Particularly, the rival kinetic models with *m* = *n* in Equation (1) cannot be discriminated under the influence of purely external diffusion limitations since the concentration dependence of the external effectiveness is nearly equal for all rate equations with *m* = *n* (Figure 5). Another limitation of the developed discrimination approach is the impossibility of distinguishing between the kinetic models based on the Langmuir–Hinshelwood and Eley–Rideal mechanisms. For instance, the rate equation $-r\left(C_{A}\right)=k\cdot K\cdot \frac{{\left(C_{A}\right)}^{m}}{{\left(1+K\cdot C_{A}\right)}^{n}}$ (Eley–Rideal) and Equation (1) (Langmuir–Hinshelwood) with *m* = *n* = 2 give the internal effectiveness factors differing only by the power of *K* in their analytic expressions. As a consequence, the concentration dependence of the internal effectiveness for both rate equations is identical.

The diffusion mechanism, e.g., Knudsen or molecular, by itself does not affect the intrinsic reaction rate. However, the interplay of the diffusion and reaction kinetics yields a considerable deviation in the concentration dependence of the observed process rate. The latter is quantified via the effectiveness factor. The dependence of the effectiveness factor on a reactant concentration is hardly ever addressed in the literature. However, investigating this dependence is essential for discrimination between the competing kinetic models. The corresponding discrimination can be performed for the process rate under the influence of external and internal diffusion limitations.

## 4. Conclusions

Several kinetic models based on different mechanisms of a heterogeneous catalytic process may provide identical descriptions of the experimental reaction rate. Under these circumstances, a problem arises in which model reflects the real situation and, therefore, should be selected for chemical reactor modeling. Statistical analysis of the models and estimated kinetic constants is typically used in the literature to achieve model selection. Instead of purely mathematical tools, a physically based approach may successfully be used for a discrimination purpose. This approach accounts for the effect of the diffusion limitations on the observed rate of a heterogeneous catalytic process. The effectiveness factor, which quantifies the effect of the diffusion limitations, notably deviates for various kinetic models with identical reaction rates. Such a situation bears for intraparticle and external diffusion of a reactant, as well as for the interplay between intraparticle and external diffusion.

The discrimination between the rival kinetic models may be performed by comparing theoretical and experimental dependencies of the effectiveness factor on the reactant concentration. In the case of entirely external diffusion limitations, the use of the diffusional discrimination approach is limited by the reaction order. Provided the reaction orders for various kinetic models are always positive or negative, it is practically impossible to select between the competing models since the external effectiveness factors are almost identical for all models. If both external and internal diffusion limitations affect the process rate, the dependence of the overall effectiveness factor on the mass transfer Biot number may be utilized for the kinetic model discrimination instead of the dependence on the reactant concentration.

Both the diffusion limitations in heterogeneous catalysis and the Langmuir–Hinshelwood reaction kinetics are well-known concepts for decades. However, their combination to achieve the selection between the competing kinetic models is introduced for the first time.

## Figures and Tables

**Table 1:** Dimensionless functions *r*(*u*) for various *m* and *n*.

*m*, *n*	*m* = *n* = 1	*m* = 1, *n* = 2	*m* = *n* = 2
*r*(*u*)	$\frac{{\left(1+\sigma \right)}^{2}\cdot u}{1+\sigma \cdot u}$	$\frac{{\left(1+\sigma \right)}^{2}\cdot u}{{\left(1+\sigma \cdot u\right)}^{2}}$	$\frac{{\left(1+\sigma \right)}^{2}\cdot \sigma \cdot u^{2}}{{\left(1+\sigma \cdot u\right)}^{2}}$

**Table 2:** Fitting results for various *m* and *n*.

*m*, *n*	*k* × 10^3^	*K*	*R* ^2^
*m* = *n* = 1	18.3 ± 0.5	12.1 ± 1.3	0.999
*m* = 1, *n* = 2	61.1 ± 0.9	2.5 ± 0.1	0.999
*m* = *n* = 2	17.8 ± 0.4	30.4 ± 2.4	0.999

**Table 3:** The analytic expressions for the internal effectiveness factor for various *m* and *n*.

*m*, *n*	Expression for the Internal Effectiveness Factor	Ref.
*m* = *n* = 1	$\eta_{int}=\frac{\sqrt{2\cdot D_{e}}\cdot {\left(1+K\cdot C_{As}\right)}^{2}}{L\cdot K\cdot C_{As}\cdot \sqrt{K\cdot k}}\cdot \sqrt{K\cdot C_{As}-\ln\left[1+K\cdot C_{As}\right]}$	[39]
*m* = 1, *n* = 2	$\eta_{int}=\frac{\sqrt{2\cdot D_{e}}\cdot {\left(1+K\cdot C_{As}\right)}^{2}}{L\cdot K\cdot C_{As}\cdot \sqrt{K\cdot k}}\cdot \sqrt{\ln\left[1+K\cdot C_{As}\right]+\frac{1}{1+K\cdot C_{As}}-1}$	[40]
*m* = *n* = 2	$\eta_{int}=\frac{\sqrt{2\cdot D_{e}}\cdot {\left(1+K\cdot C_{As}\right)}^{2}}{L\cdot K\cdot C_{As}\cdot \sqrt{K\cdot k}}\cdot \sqrt{1+K\cdot C_{As}-\frac{1}{1+K\cdot C_{As}}-2\cdot \ln\left[1+K\cdot C_{As}\right]}$	[18]

## Data Availability

The original contributions presented in this study are included in the article. Further inquiries can be directed to the corresponding author.
